# Disparities in physical fitness of 6–11-year-old children: the 2012 NHANES National Youth Fitness Survey

**DOI:** 10.1186/s12889-020-09510-x

**Published:** 2020-09-18

**Authors:** E. H. Guseman, R. Tanda, Z. T. Haile

**Affiliations:** 1 Diabetes Institute, Ohio University Heritage College of Osteopathic Medicine, Athens, OH USA; 2 Department of Primary Care, Ohio University Heritage College of Osteopathic Medicine, Athens, OH USA; 3 Nationwide Children’s Hospital, Columbus, OH USA; 4 Department of Social Medicine, Ohio University Heritage College of Osteopathic Medicine, Dublin, OH USA

**Keywords:** Fitness, Children, Socioeconomic disparities

## Abstract

**Background:**

Children’s physical fitness is an important predictor of metabolic health, physical function, and academic achievement. Although fitness is determined partially by heritable factors, it can be maintained and improved through regular physical activity. Because physical activity is known to vary by socioeconomic status, physical fitness may be expected to vary similarly. With this in mind, the purpose of this study was to examine disparities in physical fitness performance among a nationally-representative sample of 6–11 year-old children living in the United States.

**Methods:**

We conducted secondary analysis of physical fitness data of children ages 6–11 years (*n* = 686) from the nationally representative National Health and Nutrition Survey (NHANES) Youth Fitness Survey (NNYFS) 2012. We estimated sex-stratified weighted means of four fitness performance tests: cardiorespiratory endurance, upper-, lower-, and core-muscular strength. The weighted mean for each fitness assessment was compared by income groups (federal income to poverty ratio – FIPR) accounting for complex sampling design and adjusting for age.

**Results:**

Income disparities in physical fitness performance were evident among girls but not among boys. Girls from lower income groups (< 130% FIPR and 130–349% FIPR groups) showed significantly lower cardiorespiratory endurance and core muscle strength compared to those from the highest income group (≥ 350% FIPR).

**Conclusion:**

These findings highlight the need to support health-promoting physical activity among girls from disadvantaged backgrounds prior to the adolescent period.

## Background

Physical fitness among children is strongly related to metabolic health, physical function, and academic achievement [1–4]. Evidence from several large studies, including the European Youth Heart Study [5] and others [6–9] indicate that both cardiorespiratory and musculoskeletal fitness (e.g., muscular strength and muscular endurance) are vital to ensuring lifelong physical activity (PA) participation and long-term cardiovascular, musculoskeletal, and mental health. Although physical fitness is determined in part by heritable factors, it can be improved through regular engagement in PA [10–12].

Despite significant research efforts in the past two decades, childhood obesity remains a significant health concern [13]. Current estimates suggest that overall, 17.8% of U.S. children and adolescents have obesity (BMI ≥ 95th percentile), with prevalence increasing from 11.6% of 2–5-year-old children to 20.6% of 12–19-year-old adolescents [13, 14]. Further, the prevalence of severe obesity is highest among children living outside of metropolitan areas [13]. Nationally, children with excess body weight are about 25% more likely to live rural communities as opposed to urban areas [15]. Although PA represents one of the cornerstone approaches for weight maintenance, recent studies suggest that only one in seven elementary school children achieves the recommended amount of PA [16] and that girls (compared to boys) and children with obesity (compared to normal weight) are less likely to meet these recommendations [16, 17]. Barriers to PA engagement have been shown to vary according to socioeconomic status and indeed, disparities in PA according to SES have been widely noted [18]. As such, disparities in physical fitness may also be expected.

Several examinations of FITNESSGRAM data collected within school systems support the notion that socioeconomic status (SES) and neighborhood characteristics are important determinants of physical fitness among children of all ages [19–23]. State-level data from Georgia demonstrate clear disparities in fitness at the school level, where schools with less participation in the free-and-reduced school lunch program was associated with more prevalent achievement of the “healthy fitness zone” for both body mass index (BMI) and aerobic fitness; importantly, these differences varied between elementary school, middle school, and high school [19]. Similar results have been found with school-level FITNESSGRAM data in Texas [23] and California [22]. With these relationships in mind, the objective of the current study was to assess disparities in physical fitness performance among a nationally-representative sample of 6–11-year-old children who participated in the 2012 National Health and Nutrition Examination Survey (NHANES) National Youth Fitness Survey (NNYFS).

## Methods

### Data source

The present study is based on deidentified data from the 2012 NNYFS; a detailed description of the study design and methods are available elsewhere [24]. In brief, the 2012 NNYFS was conducted by the Centers for Disease Control and Prevention’s National Center for Health Statistics, to obtain data on physical activity and fitness levels of US youth aged 3 to 15 years. Similar to NHANES, the NNYFS population included a stratified, multistage probability sample representative of the civilian noninstitutionalized population in the US. The survey included interview followed by a physical fitness tests and the body measurements conducted by trained examiners. NNYFS procedures include automated quality control checks for each assessment, which include pop-up reminders prior to data entry; these are detailed in the referenced procedures manual [24]. Methods relevant to the current study are described briefly below.

### Participants

Participants included in this analysis were children who participated in the NNYFS and were aged 6–11 years at time of data collection. Participant characteristics were obtained via parent/guardian proxy report and included a brief health history, race/ethnicity, and other demographics. Physical measures included height, weight, and calculated body mass index (BMI) according to standard NHANES procedures; age- and sex-specific BMI percentile was determined using the 2000 CDC growth charts. The current study was ruled exempt according to the researchers’ Institutional Review Board because it was a secondary analysis of publicly available data (IRB #19-E-226).

#### Measures

Measures of fitness included in the NNYFS 2012 varied according to child age between 3 and 15 years. These included core muscle strength (plank), lower body muscle strength (LBMS), upper body muscle strength (hand grip), and aerobic fitness. A dietary recall was also completed for this age range. Only core muscle strength, LBMS, upper body muscle strength, and aerobic fitness completed by children aged 6–11 years were included in our analysis.

#### Cardiorespiratory fitness

Aerobic fitness was assessed via maximal treadmill test and expressed as treadmill time to exhaustion (seconds). NNYFS staff demonstrated use of the treadmill for each participant prior to allowing participants to familiarize themselves with walking on the treadmill. The protocol for all 6–11-year-old children progressed in a graded fashion with progressive increases in speed and/or grade until voluntary exhaustion. Children were encouraged to exercise as long as possible and to notify examiners if they experienced any pain, dizziness, or nausea. The treadmill protocol included a 1-min warm-up walk and subsequent stages progressed in 2-min increments according to a protocol varying by age; the full protocol has been published elsewhere [25]. Test outcome was reported as maximal treadmill time (seconds) by NNYFS. The treadmill used in this protocol (Quinton TM55) was calibrated for speed, incline, and heart rate weekly. We created age- and sex-specific z-scores for maximal endurance time to account for expected variation in endurance capacity and in order to control for the differing treadmill protocols by age categories.

#### Muscular fitness

The NNYFS isometric grip strength protocol was used to estimate upper body strength. This protocol utilizes a digital hand dynamometer (Model 5401; Takei Scientific Instruments Co., Ltd., Niigata-City, Niigata-Pref, Japan). Participants were asked to maximally squeeze the hand dynamometer, adjusted for hand size, alternately three times in each hand. Values are reported in pounds of force created from the sum of the highest value from each hand [24]. Data were available for children aged 6–15 years only; children aged 6–11 years were included in this analysis. Each participant had three trials for the grip strength test in the NNYFS. Grip strength is known to be strongly correlated with body weight [26, 27]. Therefore, we report the grip strength relative to body weight (kg) (relative hand grip strength). Upper body muscle strength was also assessed via modified pull-up; these data are not included in the present analysis.

Assessment of LBMS was completed using a hand-held tension dynamometer to assess maximal isometric knee extension force in the sitting position. Body position was maintained using a chair built specifically for the NNYFS assessments, which included straps to secure the participants’ hips, thigh, and upper body. Participants pushed their legs as hard as possible against a strap passed through the dynamometer and around the chair; participants performed three repetitions alternately with each leg and the highest values from each leg were summed for analysis [24]. Similar to grip strength, each participant had three trials for each leg during the fitness test. LBMS is also highly correlated with body weight [26]; therefore, we report the LBMS relative to child’s body weight (kg).

Core muscle endurance (trunk and pelvis) was assessed by plank hold. Participants started lying prone on the floor and pushed up into plank position with the arms resting on the forearms and weight balanced on the toes. Children were directed to hold the position as long as possible with their back straight and without the stomach dropping or hips rising up [24]. Plank hold performance has moderate negative correlations with child’s height and body weight [26, 28] and a positive correlation with cardiorespiratory endurance. Plank hold performance was found to be linearly correlated to children’s weight status in this cohort [26, 27], we normalized the value for child’s body mass index (BMI: kg/m^2^).

#### Household income and child’s age

The NNYFS collected information about participating youths’ household income, federal income-poverty ratio (FIPR) and age in years. The FIPR is calculated by dividing family income by a poverty threshold that is specific to family size. As such, using FIPR as an indicator of SES automatically adjusts for the number of people in the household. Federal assistance programs, including the free-and-reduced school lunch program, HeadStart, and supplemental nutrition assistance program (SNAP) use FIPR to determine program eligibility. For income categories, we used family income of less than 130% FIPR (equivalent to eligibility for free-and-reduced school lunch) as low income, between 130 and 349% FIPR as middle income, and greater than 350% FIPR as high income. Child’s sex was dichotomized as male or female and age at time of screening is expressed in years.

### Data analysis

Descriptive statistics are reported using frequency tables and group differences are estimated using Rao-Scott chi-square test. Mean and standard errors are reported for all fitness tests by sex and household income category using PROC SURVEYMEANS procedures. With the exception of the treadmill test, which is adjusted for age according to the protocol, least square means and standard errors for the mean were estimated using PROC SURVEYREG procedures after controlling for age. Between-group differences were evaluated by computing t-statistics at *p* < 0.05 significance using the PROC SURVEYREG procedure. Plank hold performance and LBMS values were square root transformed because of skewed distributions. Linear and quadratic trends for income gradient were assessed using orthogonal polynomial contrasts with significance accepted at *p* < 0.05. Sample weights that account for the unequal probabilities of selection, oversampling, and nonresponse and complex survey design were included in all analyses. All statistical analyses were conducted using SAS University Edition (SAS Institute, Inc., Cary, NC).

## Results

Of the 732 children ages 6 to 11 years participated in the 2012 NNYFS, 686 children had complete data for household income and were included in the current study. However, sample size used for analysis of each test varied due to missing data and/or extreme performance values. Table 1 shows characteristics of children included in the analyses. Girls were more often in the lowest income household group while more boys were in the middle-income household group (*p* = 0.012). Income differed by BMI percentile such that a greater proportion of overweight and obese children were in the middle-income group (*p* < 0.031).

**Table 1:** Sample characteristics of children ages 6–11 years old who participated in NHANES Youth Fitness Survey in 2012. *N* = 686

	Total	< 130% FIPR	130–349% FIPR	≥ 350% FIPR	*p*
	n (%)^a^	n (%)^a^	n (%)^a^	n (%)^a^	
Total	686 (100.0)	258 (33.6)	251 (37.2)	117 (29.1)	
Sex					*0.021*
Male	337 (51.3)	114 (47.2)	146 (58.2)	77 (47.2)	
Female	349 (48.7)	144 (52.8)	105 (41.8)	100 (52.8)	
BMI percentile^b^					*0.031*
< 85th	427 (64.3)	171 (67.4)	135 (55.9)	121 (71.5)	
85th – 95th	119 (16.5)	39 (15.1)	51 (19.4)	29 (14.4)^c^	
≥ 95th	139 (19.1)	48 (17.4)	64 (24.6)	27 (14.2)^c^	

Abbreviations: *FIPR* family income to poverty ratio, *P* values are obtained using Rao-Scott Chi-Squared test

^a^ Unweighted n (weighted percentage). Note: Weighted percentages were estimated by applying appropriate sampling weights and by using Taylor series linearization to account for complex sampling design

^b^ Frequency based on *n* = 685 due to missing value in body weight

^c^ Standard errors for percentage may be unstable due to small sample size

### Maximal endurance time on a treadmill

**Fig. 1:**
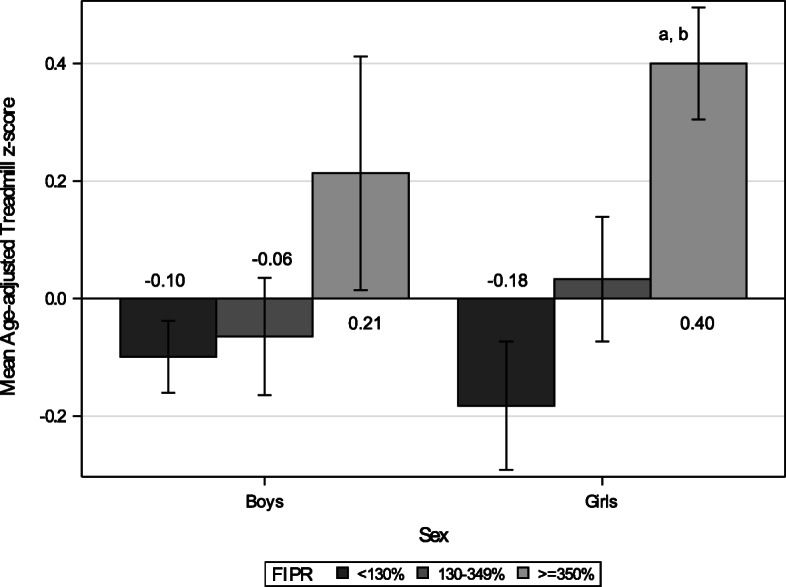
Mean Age-adjusted Treadmill z-score by Income. *N* = 635 (Boys *n* = 306, Girls *n* = 329). The number within or adjacent to each bar indicates the mean value of the group. Each error bar indicates 95% confidence interval for the mean. **a** = significant difference between low (FIPR < 130%) and high (FIPR > = 350%) income groups, controlling for child’s age (*p* = 0.004). **b** = significant difference between middle (FIPR 130–349%) and high-income groups, controlling for child’s age (*p* = 0.020)

Of the 682 participants who completed the modified treadmill test, household income data were available only among 640 children. Five data points were outside of 4 standard deviations from the mean (or less than 120 s), which left 635 children available for analysis. Compared to girls from the high-income group, girls from low- and middle-income groups fared significantly worse in the treadmill performance after controlling child’s age (*p* = 0.004 and *p* = 0.020 respectively; Fig. 1). There was a significant linear trend by income groups among girls (*p* = 0.010) but not among boys (*p* = 0.181).

### Plank hold

**Fig. 2:**
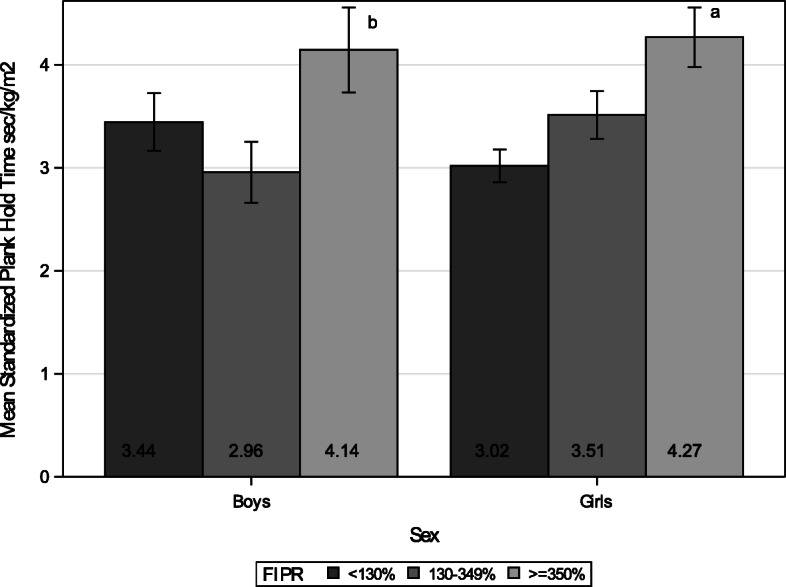
Mean Plank Hold Time Standardized for BMI (sec/kg/m2) by Income. *N* = 676 (Boys *n* = 331, Girls *n* = 345). The number within each bar indicates the least squares mean value of the group. Each error bar indicates 95% confidence interval for the mean and was estimated after controlling for child’s age. **a** = significant difference between low (FIPR < 130%) and high (FIPR > = 350%) income groups, controlling for child’s age (*p* = 0.002). **b** = significant difference between middle (FIPR 130–349%) and high-income groups, controlling for child’s age (*p* = 0.029)

Boys from the middle-income group had significantly poorer plank hold performance than those from the high-income group after controlling for child’s age (*p* = 0.029 Fig. 2). For example, a 10-year-old boy with BMI of 16.5 kg/m^2^ (50th percentile for age and sex) from the middle-income group could only hold for 46 s on a plank position while a boy from the high-income group could hold for 65 s on a plank. There was a significant quadratic trend for plank performance by income groups among boys (*p* = 0.032). On the other hand, among girls, those from the low-income group performed significantly shorter time in plank hold than those from the high-income group after controlling for child’s age (*p* = 0.002; Fig. 2). This can be translated as a 10-year-old girl with 50th percentile BMI for age and sex (BMI of 17 kg/m^2^) from the low-income group could hold for 47 s on a plank position while a girl with the same age and BMI from the high income group could hold for 65 s on a plank. There was a significant linear trend for plank performance among girls by income groups (*p* = 0.005).

### Relative grip strength

**Fig. 3:**
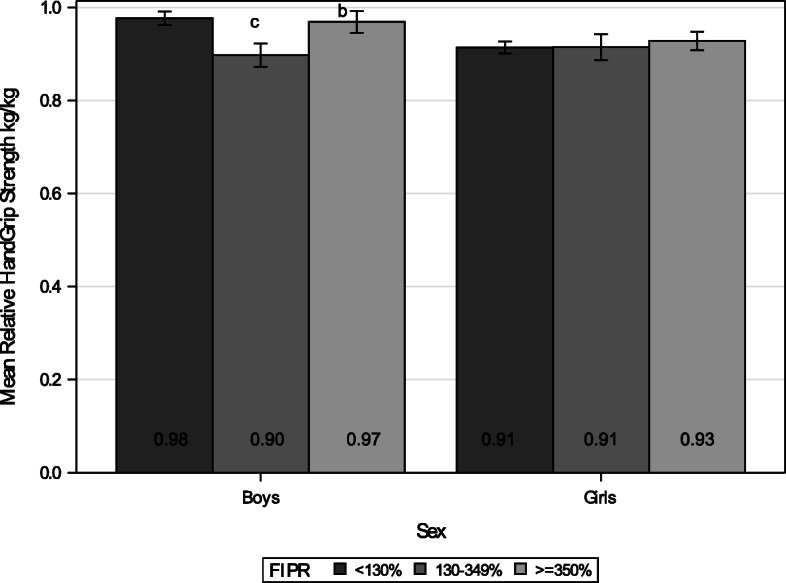
Mean Relative Handgrip Strength to Body Weight (kg/kg) by Income. *N* = 679 (Boys *n* = 334, Girls n = 345). The number within each bar indicates the least squares mean value of the group. Each error bar indicates 95% confidence interval for the mean and was estimated after controlling for child’s age. **b** = significant difference between middle and high (FIPR > = 350%) income groups, controlling for child’s age (*p* = 0.047). **c** = significant difference between low (FIPR < 130%) and middle (FIPR 130–349%) income groups, controlling for child’s age (*p* = 0.009)

Boys from the middle-income group had significantly lower handgrip performance compared to those from low- and high-income groups after controlling for child’s age (*p* = 0.009 and *p* = 0.047, respectively; Fig. 3). This could translate as a 10-year-old boy weighing 31 kg from the middle-income group performing with approximately 2 kg less grip strength than the boy with the same age and weight from the high-income group or with 2.2 kg less grip strength than the one from the low-income group. There were significant linear and quadratic trends by income groups (*p* = 0.029 and *p* = 0.015, respectively).

### Relative leg extension force

**Fig. 4:**
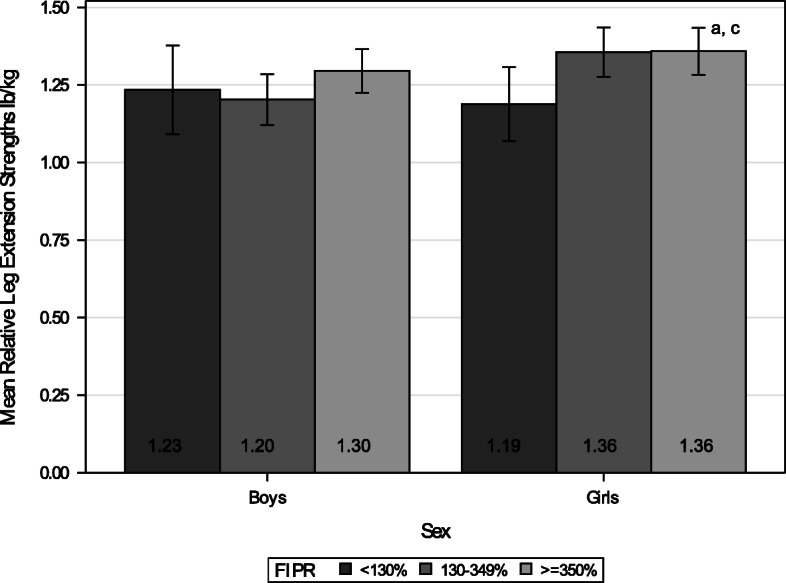
Mean Relative Leg Extension Strength to Body Weight (lb/kg) by Income. *N* = 675 (Boys *n* = 330, Girls *n* = 345). The number within each bar indicates the least squares mean value of the group. Each error bar indicates 95% confidence interval for the mean and was estimated after controlling for child’s age. **a** = significant difference between low and high (FIPR > = 350%) income groups, controlling for child’s age (*p* = 0.023). **c** = significant difference between low (FIPR < 130%) and middle (FIPR 130–349%) income groups, controlling for child’s age (*p* = 0.017)

Girls from the low-income group performed significantly worse in leg extension test than those from middle- and high-income groups after controlling for child’s age (*p* = 0.017 and *p* = 0.023, respectively; Fig. 4). This can translate as a 10-year-old girl weighting 32 kg from the low-income group being able to exert approximately 6 pounds less force with their legs than a girl of the same age and body weight from either the middle- or high-income group. There was a significant linear trend by income groups (*p* = 0.023).

## Discussion

Using a nationally representative sample of school age children from the NNYFS 2012, we found household income disparities in physical fitness. Importantly, these disparities were much more evident among girls and were found in both cardiorespiratory endurance and muscular endurance. Cardiorespiratory endurance was significantly lower among girls from lower income households compared to those from the highest income households. In addition, plank hold performance was lower among girls from the lowest income households compared to peers from the highest income households.

Plank performance among girls differed by income groups in a linear fashion, such that the lowest performance was found between the low- and high-income groups, while the difference between the middle- and high-income groups was not statistically significant. Among boys, the difference lay between the middle- and high-income groups, as evidenced by the significant quadratic trend. This may be explained in part by variation in weight status, as an inverse relationship between BMI and plank performance has been shown previously, including among children participating in the NNYFS 2012 [26, 27]. However, our analysis was adjusted for BMI. Data from Canada suggest that physical literacy – the assessment of which includes both plank hold and hand grip strength – is associated with attainment of physical activity guidelines [29]. Although PA is not included in our analysis, previous work has shown clear disparities in PA according to SES [18, 30–32]. As such, engagement in PA may explain some of the disparity shown here. It should be noted that plank hold is a measure of muscular endurance, rather than muscular strength, [24] and differences in muscular strength according to income were not found in this study.

We also found differences in cardiorespiratory fitness – or aerobic endurance – according to income level among girls and not among boys. Similar disparities in cardiorespiratory fitness were found among 9–19-year-old children in St. Louis, MO, such that fewer girls achieved the Healthy Fitness Zone in the 1-mile run; however, other details on sex differences were not provided for this sample and SES data were not provided [21]. In Georgia, broader SES-related disparities in cardiorespiratory fitness were found among girls than boys, and similar results were found in a national study of FITNESSGRAM data published in 2016 [20]. Importantly, the latter study found that minority status was not associated with cardiorespiratory fitness after controlling for SES. Though the NNYFS collected data on race and ethnicity, these data are not included in the present study due to small cell sizes that preclude meaningful interpretation of the data.

Our results are consistent with previous studies that have shown similar outcomes using more localized samples, though these primarily examined only cardiovascular fitness. A cross-sectional study using the 2010–2012 physical fitness data collected from children in 5th, 7th, and 9th grade in California public and charter schools reported that children eligible for free or reduced-price school meals had significantly lower composite fitness scores compared to those from higher income households, after adjusting for age and sex [33]. More recent FITNESSGRAM data from a single metropolitan area in California did allow for comparison of individual components of fitness; in this case, increased free-and-reduced lunch participation was associated with lower aerobic capacity and with poorer body composition [22]. Similarly, data from the Wisconsin Partnership for Childhood Fitness indicated that children in 6th grade from lower income schools had significantly lower cardiovascular fitness compared to those from higher income schools [34]. None of these studies examined sex-specific income disparities and in the latter two income was only presented at the school level rather than individual household level. Our findings strengthen the literature in this area, as we were able to examine sex-specific and individual household income disparities using a national sample.

This study is not without limitations. The cross-sectional nature of the study prohibits assessment of longitudinal changes in cardiorespiratory and muscular strength. Second, due to the relatively small sample size of the participants in the NNYFS 2012, we were unable to assess the influence of race/ethnicity or for interactions among variables. The field tests for muscular fitness and treadmill test for cardiorespiratory fitness chosen by the NNYFS differ from those children encounter for fitness assessments conducted within the school system. While these tests are all valid for elementary-aged children, differences in familiarity to the use of equipment, motivation for better performance, and variability in physical growth and maturity of each child likely contribute error. Finally, we did not adjust our analyses for physical activity because of error inherent in the use of parent-proxy report for children’s physical activity. These limitations all may have contributed to the relatively large confidence intervals observed in our results.

Nevertheless, this study has several notable strengths, which include the use of nationally representative data to describe the relationships between family characteristics and physical fitness. The use of family income to poverty ratio rather than annual household income to establish income categories allowed us to implicitly adjust for family size. Finally, we were able to include measures of muscular fitness in addition to cardiorespiratory fitness and were able to adjust these variables for body size, removing some of the influence of weight status on physical fitness. Our findings highlight socioeconomic disparities in muscular and cardiorespiratory fitness, particularly among girls, prior to the decline in habitual PA that is typically seen around puberty. As such, there are important implications for early intervention to support development of physical fitness among children from low SES groups, particularly among young girls.

## Conclusion

Disparities previously shown in physical activity according to family income are also present when measuring physical fitness among school age children. These disparities seem to be more pronounced in weight-bearing activities and among girls than boys and vary according to family income and weight status. Our results suggest disparities in physical fitness among lower income girls already exist before girls undergo the rapid decline of physical activity during adolescence. These findings highlight the need to support health-promoting physical activity among girls from disadvantaged backgrounds prior to the adolescent period.

## Data Availability

The datasets used and/or analyzed during the current study are publicly available from the Centers for Disease Control.

## Abbreviations

NHANES: National Health and Nutrition Examination Survey
NNYFS: NHANES National Youth Fitness Survey
SES: Socioeconomic Status
PA: Physical activity
MVPA: Moderate-to-vigorous physical activity
BMI: Body mass index
LBMS: Lower body muscular strength
FIPR: Federal income-to-poverty ratio

## References

[1] Donnelly JE, Hillman CH, Castelli D, Etnier JL, Lee S, Tomporowski P (2016). Physical activity, fitness, cognitive function, and academic achievement in children: a systematic review. Med Sci Sports Exerc.

[2] Lobelo F, Pate RR, Dowda M, Liese AD, Daniels SR. Cardiorespiratory fitness and clustered cardiovascular disease risk in U.S. adolescents. J Adolesc Health [Internet]. 2010 ;47(4):352–359. Available from: http://www.ncbi.nlm.nih.gov/pubmed/20864004 [cited 2011 Nov 10]. 10.1016/j.jadohealth.2010.04.012 20864004

[3] Katzmarzyk P, Malina R, Bouchard C (1999). Physical Activity, Physical Fitness, and Coronary Heart Disease Risk Factors in Youth: The Quebec Family Study. Prev Med (Baltim) [Internet].

[4] Guseman EH, Cauffman SP, Tucker JM, Smith L, Eisenmann JC, Stratbucker W. The association between measures of fitness and metabolic health in treatment-seeking youth with obesity. Metab Syndr Relat Disord. 2017;15(3):107–11. 10.1089/met.2016.0094. Epub 2016 Nov 21. 27869528

[5] Hurtig-Wennlöf A, Ruiz JR, Harro M, Sjöström M (2007). Cardiorespiratory fitness relates more strongly than physical activity to cardiovascular disease risk factors in healthy children and adolescents: the European youth heart study. Eur J Cardiovasc Prev Rehabil.

[6] Kvaavik E, Klepp K-I, Tell GS, Meyer HE, Batty GD (2009). Physical fitness and physical activity at age 13 years as predictors of cardiovascular disease risk factors at ages 15, 25, 33, and 40 years: extended follow-up of the Oslo youth study. Pediatrics [Internet].

[7] Blair SN, Cheng Y, Holder JS. Is physical activity or physical fitness more important in defining health benefits? Med Sci Sports Exerc [Internet]. 2001 ;33(6 Suppl):S379–S399; discussion S419–20. Available from: http://www.ncbi.nlm.nih.gov/pubmed/11427763. 10.1097/00005768-200106001-00007 11427763

[8] Schmidt MD, Magnussen CG, Rees E, Dwyer T, Venn AJ. Childhood fitness reduces the long-term cardiometabolic risks associated with childhood obesity. Int J Obes [Internet] 2016;40(7):1134–1140. Available from: 10.1038/ijo.2016.61. 10.1038/ijo.2016.61 27102049

[9] Ekelund U, Anderssen SA, Froberg K, Sardinha LB, Andersen LB, Brage S. Independent associations of physical activity and cardiorespiratory fitness with metabolic risk factors in children: the European youth heart study. Diabetologia [Internet]. 2007;50(9):1832–1840. Available from: http://www.ncbi.nlm.nih.gov/pubmed/17641870 [cited 2012 Jul 22]. 10.1007/s00125-007-0762-5 17641870

[10] Zaqout M, Vyncke K, Moreno LA, De Miguel-Etayo P, Lauria F, Molnar D (2016). Determinant factors of physical fitness in European children. Int J Public Health.

[11] Schutte NM, Nederend I, Hudziak JJ, de Geus EJC, Bartels M (2016). Differences in adolescent physical fitness: a multivariate approach and meta-analysis. Behav Genet.

[12] Bouchard C, An P, Rice T, Skinner JS, Wilmore JH, Gagnon J, et al. Familial aggregation of VO2max response to exercise training: results from the HERITAGE Family Study. J Appl Physiol [Internet]. 1999 ;87(3):1003–1008. Available from: http://www.ncbi.nlm.nih.gov/pubmed/10484570 [cited 2012 Dec 6]. 10.1152/jappl.1999.87.3.1003 10484570

[13] Ogden CL, Fryar CD, Hales CM, Carroll MD, Aoki Y, Freedman DS. Differences in obesity prevalence by demographics and urbanization in US children and adolescents, 2013-2016. JAMA. 2018;319(23):2419–29. 10.1001/jama.2018.7270. 10.1001/jama.2018.5158 PMC6393914 29922826

[14] Hales CM, Fryar CD, Carroll MD, Freedman DS, Ogden CL. Trends in obesity and severe obesity prevalence in US youth and adults by sex and age, 2007-2008 to 2015-2016. Jama [Internet]. 2018:1–3 Available from: http://jama.jamanetwork.com/article.aspx?doi=10.1001/jama.2018.3060. 10.1001/jama.2018.3060 PMC5876828 29570750

[15] Lutfiyya MN, Lipsky MS, Wisdom-Behounek J, Inpanbutr-Martinkus M (2007). Is rural residency a risk factor for overweight and obesity for U.S. children?. Obesity.

[16] Hubbard K, Economos CD, Bakun P, Boulos R, Chui K, Mueller MP, et al. Disparities in moderate-to-vigorous physical activity among girls and overweight and obese schoolchildren during school- and out-of-school time. Int J Behav Nutr Phys Act [Internet] 2016;13(1):1–8. Available from: 10.1186/s12966-016-0358-x. 10.1186/s12966-016-0358-x PMC4802912 27000400

[17] Fakhouri THI, Hughes JP, Brody DJ, Kit BK, Ogden CL. Physical activity and screen-time viewing among elementary school-aged children in the United States from 2009 to 2010. JAMA Pediatr [Internet]. 2013 ;167(3):223–229. Available from: http://www.ncbi.nlm.nih.gov/pubmed/23303439 [cited 2014 Apr 5]. 10.1001/2013.jamapediatrics.122 23303439

[18] Katzmarzyk PT, Denstel KD, Beals K, Carlson J, Crouter SE, McKenzie TL (2018). Results from the United States 2018 report card on physical activity for children and youth. J Phys Act Health.

[19] Bai Y, Saint-Maurice PF, Welk GJ (2017). Fitness trends and disparities among school-aged children in Georgia, 2011-2014. Public Health Rep.

[20] Bai Y, Saint-Maurice PF, Welk GJ, Allums-Featherston K, Candelaria N (2016). Explaining disparities in youth aerobic fitness and body mass index: relative impact of socioeconomic and minority status. J Sch Health.

[21] Ruth Clark B, Leanne White M, Royer NK, Burlis TL, DuPont NC, Wallendorf M (2015). Obesity and aerobic fitness among urban public school students in elementary, middle, and high school. PLoS One.

[22] Kahan D, McKenzie TL (2017). School and neighborhood predictors of physical fitness in elementary school students. J Sch Health.

[23] Welk GJ, Ihmels M, Seeger C, Meredith MD (2010). Distribution of health-related physical fitness in Texas youth: a demographic and geographic analysis. Res Q Exerc Sport.

[24] Borrud L, Chiappa MM, Burt VL, Gahche J, Zipf G, Johnson CL, et al. National Health and Nutrition Examination Survey: national youth fitness survey plan, operations, and analysis, 2012. Vital Heal Stat. 2014;(163):1–24. 24709592

[25] National Center for Health Statistics (2012). National Youth Fitness Survey (NYFS)- Treadmill Examination Manual.

[26] Ervin RB, Fryar CD, Wang C-Y, Miller IM, Ogden CL (2014). Strength and body weight in US children and adolescents. Pediatrics [Internet].

[27] Laurson KR, Saint-Maurice PF, Welk GJ, Eisenmann JC (2017). Reference Curves for Field Tests of Musculoskeletal Fitness in U.S. Children and Adolescents: The 2012 NHANES National Youth Fitness Survey. J strength Cond Res [Internet].

[28] Strand SL, Hjelm J, Shoepe TC, Fajardo MA (2014). Norms for an isometric muscle endurance test. J Hum Kinet.

[29] Belanger K, Barnes JD, Longmuir PE, Anderson KD, Bruner B, Copeland JL, et al. The relationship between physical literacy scores and adherence to Canadian physical activity and sedentary behaviour guidelines. BMC Public Health. 2018;18(Suppl 2):1042. 10.1186/s12889-018-5897-4. 10.1186/s12889-018-5897-4 PMC6167767 30285783

[30] Barr-Anderson DJ, Flynn JI, Dowda M, Taverno Ross SE, Schenkelberg MA, Reid LA (2017). The modifying effects of race/ethnicity and socioeconomic status on the change in physical activity from elementary to middle school. J Adolesc heal [Internet].

[31] Trost SG, Mccoy TA, Vander Veur SS, Mallya G, Duffy ML, Foster GD (2013). Physical activity patterns of inner-city elementary schoolchildren. Med Sci Sports Exerc.

[32] Kelly EB, Parra-Medina D, Pfeiffer KA, Dowda M, Conway TL, Webber LS (2010). Correlates of physical activity in black, Hispanic, and white middle school girls. J Phys Act Health [Internet].

[33] Jin Y, Jones-Smith JC (2015). Associations between family income and children’s physical fitness and obesity in California, 2010-2012. Prev Chronic Dis [Internet].

[34] Bowser J, Martinez-Donate AP, Carrel A, Allen DB, Paul MD (2016). Disparities in fitness and physical activity among children. Wis Med J [Internet].

